# Determinants and reasons for switching anti-retroviral regimen among HIV-infected youth in a large township of South Africa (2002–2019)

**DOI:** 10.1186/s12981-022-00453-4

**Published:** 2022-06-28

**Authors:** Anita Kabarambi, Sheila Balinda, Andrew Abaasa, Dolphina Cogill, Catherine Orrell

**Affiliations:** 1 Medical Research Council/Uganda Virus Research Institute and London School of Hygiene and Tropical Medicine, Uganda Research Unit, Entebbe, Uganda; 2 London School of Hygiene and Tropical Medicine, London, UK; 3 Desmond Tutu HIV Centre, Department of Medicine and Institute for Infectious Disease and Molecular Medicine, Faculty of Health Sciences, University of Cape Town, Cape Town, South Africa

**Keywords:** Drug switch, Antiretroviral therapy, Young people, South Africa, Reasons for switch

## Abstract

**Background:**

There are limited data exploring antiretroviral therapy (ART) changes and time to change among South Africa young people living with HIV/AIDS.

**Objective:**

We describe the time to first drug switch, which includes ART regimen change (three drug switch) and substitutions (single drug switch). We describe common reasons for ART switch among young people aged 10 to 24 years in South Africa.

**Methods:**

We conducted a retrospective cohort study at a primary health care clinic in Cape Town, South Africa, providing ART to HIV-infected adolescents and adults since 2002. Those aged 10 to 24 years at ART initiation, who accessed care clinic between September 2002 and April 2019. Data was retrieved from electronic information systems: ART regimens, ART changes, dates for initiation or stop of each drug/regimen, laboratory results (viral loads, haemoglobin, liver enzyme results, and creatinine to support the reason for ART switch. From written records, we abstracted reason for single drug switch or regimen change, as well as socio demographic and clinical data. We fitted cox regression models to determine factors associated with ART switch (Having a change in one or more drugs in ART combination) and the rate of occurrence.

**Results:**

Of 2601 adolescents included, 605 (24.9%) adolescents switched ART over 5090.5 person years at risk (PYAR), a rate of 11.9 /100PYAR. Median follow-up time was 4.4 (± 3.2) years. At multivariable analysis, the older age group was protective of the risk of ART switch: adjusted Hazard Ratio [aHR] 0.78, 95% CI 0.62–0.98, transfer status [transferred out 1.42 [1.11–1.82]. The hazard of ART switch increased with more severe HIV-disease at ART start, as observed by increasing WHO clinical stage or reduced CD4 count at baseline. The primary reasons for ART switch were side effects (20.0%), virological failure (17.9%) and formulation switch (27.8%). Others reasons included pregnancy, Hepatitis B, tuberculosis and psychosis.

**Conclusion:**

ART switches are frequent and occur at a consistent rate across 7.5 years from initiation. The main reasons for ART switch were virological failure and drug side effects.

## Introduction

Adolescents and young people represent a growing share of people living with HIV worldwide. In 2019 alone, 460,000 young people between the ages of 10 to 24 were newly infected with HIV, of whom 170,000 were adolescents between the ages of 10 and 19 [1]. AIDS is the leading cause of death among young people (aged 10–24) in Africa, and second leading cause globally [2]. Despite the introduction of highly active antiretroviral therapy (ART) in 1996 and more effective biomedical prevention strategies, HIV/AIDS remains a global pandemic [3].

The World Health Organisation’s (WHO) test and treat strategy recommends that all people newly diagnosed with HIV start ART immediately, regardless of disease stage or CD4 count [4]. Starting ART earlier in the disease process increases lifelong exposure to ART. All PLHIV will be exposed to multiple ART regimens in their lifetime, due to drug changes required for toxicity, treatment failure or to update/simplify their regimen. Children and adolescents started on ART will require successful ART for more years than any adult population. Systematic reviews have shown that people living with HIV (PLWHIV) will nearly all have changes in their ART at a some point during their follow up [5, 6]. ART changes in low and middle income countries (LMICs) are limited by available treatment options [7]. There is a critical need, especially for younger people or adolescents starting ART, to conserve the use of each drug and each regimen, especially in resource-limited settings where treatment options remain limited.

To date there are limited data exploring reasons for ART drug switches (single drug substitutions or whole regimen changes) and time to change among South African adolescents with HIV/AIDS. The aims of this study are to describe the time to, and common reasons for, ART switch among young people aged 10 to 24 years in South Africa.

## Methods

### Study design

This was a retrospective cohort study using ART data collected at the Hannan Crusaid Treatment Centre from 2002 to 2019. We used the data collected to explore reasons for ART drug switch, time to first switch and reason for change among adolescents.

### Setting

This study was conducted at Hannan Crusaid Treatment Centre (HCTC) in Gugulethu, Cape Town, South Africa. HCTC is a primary health care clinic that has been providing ART to HIV-infected adolescents and adults since 2002.

At the HCTC, HIV/AIDS treatment is offered according to the South African National ART guidelines [8] Between 2002 and 2008 the first-line ART regimen for those aged 10 to 24 years was either stavudine (d4T), abacavir(ABC) or Zidovudine (AZT), in combination with lamivudine (3TC), and either efavirenz (EFV) or nevirapine (NVP). From 2010, first line ART at the Hannan Crusaid Clinic in ART-naive adolescents and adults comprised Truvada (TDF)/emtricitabine(FTC) and either EFV or NVP. Patients that had previously started regimens containing AZT or d4T were maintained on these regimens unless toxicity developed [7]. By 2013, all adolescents had been switched onto this new regimen.

### Study population

*Eligibility criteria.* All data from young HIV-positive people (aged 10 to 24 years at the time of ART initiation) who had accessed care at the HCTC HIV clinic between September 2002 and April 2019, as evidenced by any ART prescription in that period, were included in this analysis. We excluded 41 young people who did not have any ART records in their files.

### Measures

HCTC maintains electronic information systems to capture routine clinical information from patient records. The data retrieved included demographic and disease characteristics of all people aged 10 to 24 years at the time of ART initiation between September 2002 and April 2019, including age, sex, starting CD4 cell count and WHO staging at programme entry. We also retrieved ART regimens including any changes made, plus the dates for initiation and stopping of every regimen. We accessed laboratory results including viral loads, haemoglobin levels, liver enzyme tests, and creatinine levels to confirm the reason for ART switch. From written records, we abstracted reasons for switch at each time point and any other information that was missing in the electronic database.

#### Outcomes and exposure

The primary outcome variable was time to first ART switch due to any reason. We defined a drug switch as any substitution from d4T, AZT, ABC and TDF, or as a change from first-line treatment to second-line treatment regimen.

Our analysis did not consider the ART programmatic change in South Africa in 2010 to adapt the WHO recommendations to move to a TDF-based regimen as a reason for ART switch. All patients were switched to a TDF based regimen at that time.

The association between ART switch and the exposure variables, which included the age at ART initiation, sex, baseline CD4, WHO staging [9], status of ART at entry into the clinic and transfer status into the HCTC of the patient, was examined.

### Statistical analysis

The study data were captured in a Microsoft Excel sheet and transferred to Stata version 16.1 (College Station, Texas, USA) for analysis. We summarised the adolescents’ baseline socio-demographic and clinical characteristics by use of frequencies and percentage and compared them between those that switched ART regime and those that did not by use of Chi-square tests. Our primary outcome was time to first ART switch; therefore, we estimated the hazard of ART switch as total number of adolescents that experienced ART switch in the study duration divided by the total time at risk. The total time at risk was estimated as time from ART initiation to the first ART switch for adolescents that switch ART or the date of censorship for those that did not switch ART. We plotted a Kaplan Meier graph for year of ART initiation because there was a greater chance of ART switch prior to 2010.

We fitted Cox proportional hazards models at both bivariable and multivariable stages to find correlates of ART switch. At bivariable analysis, factors that attained a statistical significance, log likelihood ratio test p value less than 0.05 were considered for multivariable model. In the multivariable model, factors were removed from the model using a backward elimination retaining any factors whose log-likelihood ratio p-value for inclusion was less than 0.05. Reasons for ART regime switching and side effects were summarized using counts and percentages focusing on prior the programmatic change (2010) and after. Adjusted for variables included; sex (a priori), others were age, WHO stage, transfer status, baseline CD4 count, year of ART initiation and NNRTI/PI based regimen.

## Results

### Enrolment of young PLWHIV into the HCTC

Of the 16,946 people living with HIV enrolled at the clinic since September 2002, 2,207 (13%) were young people aged 10–24 years. Of these, 2061 were initiated on ART and were eligible to be included in this study.

### Baseline characteristics

The majority of the adolescents enrolled into our study were women, aged 18–24 years, who were ART-naïve at entry into the clinic (Table 1). Although most had early HIV-disease (WHO clinical stage 1) at ART start, over a quarter (25.7%) had later stage disease (WHO stage 3 or 4); and 52.1% had a baseline CD4 count of < 250 cells/mm^3^. Most (85.9%) commenced efavirenz-based regimens. The median time on ART was 4.4 (± 3.2) years at the time of censoring (30 April 2019).

**Table 1:** Baseline characteristics of young people living with HIV accessing HIV care services at the Hannan Crusaid Treatment Centre in Cape town South Africa (2002–2019)

Variable	Sub category	Total sample		Never switched		Switched		p-value
		n = 2,061	Percent	n = 1,456	Percent	n = 605	Percent	
Sex	Male	216	10.5	129	59.7	87	40.3	< 0.001
	Female	1,845	89.5	1,327	71.9	518	28.1	
Age (years)	10–17	212	10.3	115	54.3	97	45.7	< 0.001
	18–24	1,849	89.7	1,341	72.5	508	27.5	
WHO stage	l	1,213	58.9	1,008	83.1	205	16.9	< 0.001
	lll	318	15.4	205	64.5	113	35.5	
	lll	390	18.9	177	45.4	213	54.6	
	lV	140	6.8	66	47.1	74	2.9	
ART status at entry into the clinic	Naive	2,032	98.6	1,435	70.6	597	29.4	0.833
	Experienced	29	1.4	21	72.4	8	27.6	
Transfer status into HCTC from another clinic	Not transferred	1,643	79.7	1,114	67.8	529	32.2	< 0.001
	Transferred in	418	20.3	342	81.8	76	18.2	
Baseline CD4	< 250	1,073	52.1	577	53.8	496	46.2	< 0.001
	250–499	775	37.6	680	87.7	95	12.3	
	500 +	213	10.3	199	93.4	14	6.6	
Year of ART initiation	Before 2010	457	22.2	168	36.8	289	63.2	< 0.001
	2010 +	1,604	77.8	1,288	80.3	316	19.7	
NNRTI/PI based	NVP	239	11.6	87	36.4	152	63.6	< 0.001
	EFV	1,771	85.9	1,347	76.1	424	23.9	
	LPV/r	48	2.3	21	43.7	27	56.3	
	ATV/r	3	0.2	1	33.3	2	66.7	

### Drug changes

Overall, 605 (29.4%) young people switched their ART during their follow up. These switches are described in Table 2. Most non-nucleoside reverse transcriptase inhibitor (NNRTI) switches were to another NNRTI, or to a protease inhibitor (Table 2). Those with NVP in their regimen were more likely to switch (72/152; 47.3%), compared to EFV (116/424; 27.4%); p < 0.001. Nucleoside reverse transcriptase inhibitor (NRTI) switches were largely from d4T to another NRTI (159/242; 65.7%) (Table 2). There were few switches off TDF (26/242, 10.7%) Table 2.

**Table 2:** Initial ART and ART after switch for young people living with HIV accessing HIV care services at the Hannan Crusaid Treatment Centre in Cape town South Africa (2002–2019)

a NNRTI and PI switches					
Initial ART (NNRTI/PI)	ART after switch				Total
	NVP	EFV	LPV/r	ATV/r	
NVP	–	49	23	0	72
EFV	45	–	70	1	116
LPV/r	0	1	–	1	2
ATV/r	0	0	0	–	0
Total	45	50	93	2	190

b NRTI switches					
Initial ART (NRTI)	ART after switch				Total
	d4T	AZT	ABC	TDF	
d4T	–	97	8	54	159
AZT	17	–	2	20	39
ABC	0	4	–	14	18
TDF	2	19	5	–	26
Total	19	120	15	88	242

NVP: Nevirapine; EFV: Efavirenz; ATV/r-Atazanavir/ritonavir, LPV/r-Lopinavir/ritonavir

### Time to ART switch and proportion of switches

**Fig. 1:**
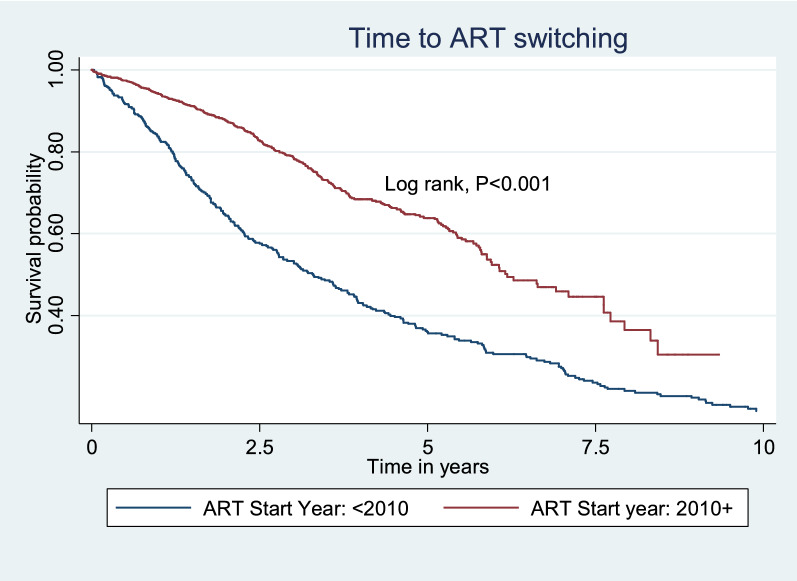
Kaplan Meier Analysis: showing the probability of not switching ART over time (in years) for young people living with HIV attending HIV care services in the Hannan Crusaid HIV Centre in Cape Town South Africa (2002–2019)

The Kaplan Meier analysis (Fig. 1) shows that there was a fairly consistent rate of switch, over each year from year 1 to year 7.5; then a slight flattening of the curve indicating fewer later switches. There was a greater chance of ART switch prior to 2010, driven by changes from d4T.

Overall, more males switched compared to females (40.3% vs 28.1%, p < 0.001) and younger people (age 10–17 years) switched versus those ages 18–24 years (45.7% vs 27.5%, p < 0.001) (Table 1). The proportion who switched ART increased with worsening illness at programme entry: by progressive WHO stage (for stage I to III) and decreasing baseline CD4 count (Table 1).

### Reasons for switch

The main categories for the 605 ART switches are described in Table 3. Prior to 2010, side effects (n = 101; 34.9%) were the most common reason for switch. The main side effects reported during this period were lipodystrophy and peripheral neuropathy (Table 4). After 2010, most switches happened due to formulation switch, namely 3TC to FTC and vice versa (n = 165; 52.2%). Side effects accounted for 6.6% of switches later in the programme.

**Table 3:** Categories for ART switch among young people living with HIV attending HIV care services in the Hannan Crusaid HIV Centre in Cape town South Africa, stratified by period of starting ART (< 2010 vs 2010 +)

Reasons for switching ART	Before 2010	2010 +	Overall
	Freq (%)	Freq (%)	Freq (%)
Formulation switch e.g. TDF/3TC to TDF/FTC	3 (1.0)	165 (52.2)	168 (27.8)
Side effects	101 (34.9)	21 (6.7)	122 (20.0)
Virological Failure	55 (19.0)	53 (16.8)	108 (17.8)
Programmatic e.g. switch from d4T to TDF	38 (13.2)	32 (10.1)	70 (11.6)
Pregnancy	54 (18.7)	13 (4.1)	67 (11.1)
Regimen simplification	6 (2.1)	22 (7.0)	28 (4.6)
Tuberculosis	19 (6.6)	8 (2.5)	27 (4.5)
Defaulted	10 (3.5)	0 (0.0)	10 (1.7)
Psychosis	1 (0.3)	0 (0.0)	1 (0.2)
Hepatitis B	0 (0.0)	1 (0.3)	1 (0.2)
Reason not specified	2 (0.7)	1 (0.3)	3 (0.5)
Total	289	316	605

**Table 4:** Details of side effects (n = 121) resulting in switch among ART experienced by young people living with HIV attending HIV care services in the Hannan Crusaid HIV Centre in Cape town South Africa prior to and after 2010

Side effects	Before 2010	2010 +	Overall
	Frequency (%)	Frequency (%)	Frequency (%)
Lipodystrophy	55 (54.5)	2 (10.0)	57 (47.1)
Peripheral neuropathy	25 (24.8)	2 (10.0)	27 (22.3)
Anaemia	12 (11.9)	4 (20.0)	16 (13.2)
Nephrotoxicity	0 (0.0)	5 (25.0)	5 (4.1)
Other	9 (8.9)	7 (35.0)	16 (13.2)
Total	101	20	121

### Bivariate analysis

At bivariate analysis, being younger (10–17 years), having later stage disease (WHO stage III or IV or CD4 cell count < 250), being on a PI-based regimen at ART initiation and being transferred in from another clinic were baseline characteristics significantly associated with ART switching (Table 5).

**Table 5:** Comparison of independent variables with ART switch among 605 young people living with HIV attending HIV care services in the Hannan Crusaid HIV Centre in Cape Town South Africa (2002–2019) that switched ART during this time

Variable	Sub category	Rate Per 100 *PYO	Unadjusted HR (95% CI)	^ƒ^LRT p-value	Adjusted HR (95% CI) for all variables	^£^p-value
Overall	–	11.9				
Sex	Male	12.8	1.00	0.612	1.00	
	Female	11.7	0.94 (0.75–0.1.18)		1.19 (0.94–1.53)	0.155
Age (years)	10–17	15.2	1.00	0.018	1.00	
	18–24	11.4	0.76 (0.61–0.95)		0.78 (0.62–0.98)	0.032
WHO stage	l	7.7	1.00	< 0.001	1.00	
	ll	13.3	1.72 (1.37–2.16)		1.34 (1.06–1.70)	0.014
	lll	17.7	2.25 (1.85–2.73)		1.73 (1.40–2.14)	< 0.001
	lV	19.4	2.46 (1.88–3.21)		2.09 (1.58–2.77)	< 0.001
ART status at entry into clinic	Naive	12.0	1.00	0.121		
	Experienced	7.5	0.60 (0.30–1.21)			
Transfer status	Not transferred		1.00	< 0.001	1.00	
	Transferred out		1.63 (1.27–2.08)		1.42 (1.11–1.82)	0.006
Baseline CD4	< 250	16.1	1.00	< 0.001	1.00	
	250–499	6.0	0.38 (0.30–0.47)		0.51 (0.40–0.66)	< 0.001
	500 +	3.3	0.21 (0.13–0.36)		0.29 (0.17–0.49)	< 0.001
Year of ART initiation	Before 2010	19.0	1.00	< 0.001	1.00	
	2010 +	8.8	0.46 (0.39–0.55)		0.97 (0.79–1.19)	0.777
NNRTI/PI based	NVP	22.5	1.00	< 0.001	1.00	
	EFV	10.1	0.45 (0.38–0.55)		0.62 (0.50–0.76)	
	LPV/r	13.5	0.59 (0.39–0.89)		0.62 (0.41–0.94)	< 0.001
	ATV/r	13.7	0.57 (0.14–2.29)		0.48 (0.12–1.97)	0.310

CI: Confidence interval, HR: Hazard ratio

^ƒ^LRT-Log likelihood test p-value

^£^p-value-Wald probability value

*PYO-Person years of Observation

### Multivariate analysis

ART switch remained associated with age in adjusted models, with older age (18–24 years versus 10–17 years) being protective against switch: adjusted Hazard Ratio [aHR] 0.71, 95% CI 0.56–0.59. Similarly, later stages of illness were increasingly associated with switching: compared to stage I, the hazard of switch was 62% higher among stage II (aHR 1.62, 1.28–2.05), 90% higher among stage III (aHR 1.90, 95% CI 1.55–2.33) and 88% higher among those with stage IV disease (aHR 1.88, 95% CI 1.43–2.47). Higher CD4 cell counts at baseline were protective of switch: compared to those with a baseline CD4 of < 250 cells/µ, the hazard of ART switch was 43% lower for those with a baseline CD4 cell count between 250 and 499 cells/µ (aHR 0.43, 95% CI 0.34–0.54) and 21% lower for those with baseline CD4 of 500 + cells/µ (aHR 0.21, 95% CI 0.12–0.36). Hazard of switch was lower for any ART agent when compared to NVP (aHR 0.62, 95% CI 0.41–0.94) (Table 5).

## Discussion

We found that at a large ART clinic in Cape town, close to 30% of young people underwent a switch in their prescribed ART over their time in care. These young people switched ART over 5090.5 person years at risk (PYAR), at a rate of 11.9/100PYAR. Median follow-up time was 4.4 (± 3.2) years. The rate of switching was constant for the first 7.5 years of follow-up and tapered slightly thereafter. The rate of ART switching was less with newer regimens offered after 2010, compared to regimens used earlier in the treatment programme. Young adolescents (ages 10–17 years) and those with lower baseline CD4 cell counts (< 250 cells/mm3) were more likely to switch their ART. This could be due to the fact that they do not have fully developed risk assessment skills, impulse control, or organizational abilities which in the long run contribute to low adherence rates and virological failure leading to drug switch [10]. Those with advanced HIV disease (WHO changes 2, 3 or 4) were also more likely to switch their ART compared to those in stage one. The majority of ART switches prior to 2010 were due to side effects (35%), however after 2010, with the advent of better tolerated treatment regimens, side effects only accounted for 6.6% of switches. After 2010, the main reason for switch was alteration of tablet formulation. Virological failure accounted for 16–19% of switches both prior to and after 2010. Pregnancy, tuberculosis, psychosis, treatment simplification and defaulting were among other reasons why young people switched ART drugs.

Previous studies looking at reasons and rate of ART switching have focused on adults. This study was novel as we examined drug changes in younger people from the age of 10 years. We also reviewed data collected over close to two decades (± 17 years) unlike previous cohorts followed over much shorter periods, of 2 years or less [11, 12]. A study conducted in 2004 found that most ART switching happened within 2 years of starting ART [17]. This was not the case for our study, where switches occurred gradually over time. Development and use of newer drugs that are better tolerated and less toxic will likely have contributed to the longer duration on ART before switching [11]. Prior work among adults with low CD4 counts, showed they were more likely to switch medication [13]; and our study confirmed this in adolescents: young people were less likely to switch ART if their CD4s were greater than 250 cells/µL at baseline.

Previous studies have noted that young people have more challenges taking their treatment mainly due to side effects, forgetting and stigma; resulting in eventual virological failure [14–16]. Our data reflect this, with almost a quarter of the young people switching ART due to side effects and a similar proportion due to virological failure. While there was a marked reduction in switches due to side effects after the introduction of Tenofovir to the South African public sector ART programme in 2010, the proportion experiencing virological failure remains the same.

There is an ongoing concern that young people may run out of options for treatment over time, as ART is lifelong therapy [17]. With close to 30%of the cohort changing their treatment due to side effects and virological failure within their first ten years on treatment, options for future decades of treatment may soon become limited. Focus on adherence support for this cohort is critical [17]. Rapid adoption of newer drugs with reduced tablet burden and fewer side-effects into ART schedules is critical, as safer medications are shown to reduce the number of switches in ART. Clinicians attending to young people living with HIV need to pay special attention to those with CD4 < 250 cells/µL, providing proactive adherence counselling and closer monitoring to support adherence, allowing longer time on simpler, safer and cheaper first-line regimens.

## Limitations

Issues revealed when sampling a single clinic are not always representative of other similar cohorts in South Africa. This clinic is however typical of provincial health services and thus does give some insight into reasons for switch, and the time to change ART, among this group.

We collected data retrospectively, using already collected data from a database and folders. We were unable to trace the reasons for ART switch in 41 young people, which may have biased our results.

## Conclusions

Our study found that ART switches among young people are frequent, but happen gradually over the first 7.5 years after ART initiation; with the main reasons for switch being virological failure and drug side effects. We recommend further research on association of ART switching in younger people with ART adherence, drug resistance and the impact of co-morbidities such as tuberculosis.

## Data Availability

The data set that was analysed for this study are available.
